# Characterization and Production of Antimicrobial Compound by *Streptomyces* Isolated From the Ant *Polyrhachis laevissima*

**DOI:** 10.1155/ijm/8925797

**Published:** 2026-05-22

**Authors:** Tuangrat Tunvongvinis, Weeyawat Jaitrong, Yudthana Samung, Chaisak Chansriniyom, Achiraya Somphong, Somboon Tanasupawat, Wongsakorn Phongsopitanun

**Affiliations:** ^1^ Department of Biochemistry and Microbiology, Faculty of Pharmaceutical Sciences, Chulalongkorn University, Bangkok, Thailand, chula.ac.th; ^2^ Office of Natural Science Research, National Science Museum, Pathum Thani, Thailand; ^3^ Department of Medical Entomology, Faculty of Tropical Medicine, Mahidol University, Bangkok, Thailand, mahidol.ac.th; ^4^ Department of Pharmacognosy and Pharmaceutical Botany, Faculty of Pharmaceutical Sciences, Chulalongkorn University, Bangkok, Thailand, chula.ac.th; ^5^ Center of Excellence in Natural Products and Nanoparticles, Chulalongkorn University, Bangkok, Thailand, chula.ac.th

**Keywords:** actinomycetes, actinomycin D, ant, antimicrobial activity, social insect, *Streptomyces*

## Abstract

Social insects such as ants, wasps, and bees have been recognized as promising sources of actinomycetes with antimicrobial potential. In a previous study, we isolated an ant‐derived *Streptomyces* strain, LKA04, from *Polyrhachis laevissima*, which exhibited potent antimicrobial activity. However, the antimicrobial compound produced by this strain had not been previously characterized. Based on phenotypic and genotypic analyses, strain LKA04 was identified as *Streptomyces parvulus*. Bioassay‐guided fractionation led to the successful isolation of the purified antimicrobial compound produced by this strain. HPLC chromatographic comparison with an in‐house database, along with NMR and mass spectrometry analyses, identified the active compound as actinomycin D. This compound exhibited broad‐spectrum antimicrobial activity against gram‐positive bacteria, including *Staphylococcus aureus*, methicillin‐resistant *Staphylococcus aureus* (MRSA), *Bacillus subtilis*, *Kocuria rhizophila*, *Enterococcus faecalis*, and *Listeria monocytogenes*. It also demonstrated activity against gram‐negative bacteria such as *Klebsiella pneumoniae* and *Acinetobacter baumannii*, as well as the yeasts *Candida albicans* and *Candida glabrata*. The highest yield of actinomycin D (217 mg/L) was achieved when *S. parvulus* LKA04 was cultured in glutamic acid–histidine–fructose mineral salts broth at 30°C under shaking conditions (180 rpm) for 14 days. This study highlights the potential of ant‐derived actinomycetes as valuable sources of antimicrobial compounds and underscores the need for continued investigation to further advance our understanding of their biosynthetic capabilities and pharmaceutical potential.

## 1. Introduction

Actinomycetes are gram‐positive, filamentous bacteria widely distributed across diverse habitats [1]. They are well known as prolific producers of antibiotics and other bioactive secondary metabolites [2]. Over the past three decades, insects in the family Hymenoptera—including ants, wasps, and bees—have been recognized as promising sources of microorganisms with the potential to produce bioactive natural products, particularly antimicrobial compounds [3]. One of the most extensively studied mutualistic relationships is that of leaf‐cutting ants, which harbor actinomycetes to protect their nests from pathogenic fungi [4]. Notably, this mutualistic interaction is not limited to leaf‐cutting ants and has also been documented in various other ant species [5]. Ants represent an underexplored source of actinomycetes, from which several novel species have recently been isolated. Examples include *Streptomyces odontomachi*, isolated from *Odontomachus simillimus* Smith (1858) [6]; *Streptomyces camponoticapitis*, isolated from the head of *Camponotus japonicus* Mayr [7]; and *Amycolatopsis camponoti*, isolated from *Camponotus vagus* [8]. These discoveries highlight the potential of ants as reservoirs of previously uncharacterized actinomycetes with biotechnological relevance. In recent years, several novel bioactive compounds have been isolated from ant‐derived actinomycetes. For example, formicolides A and B, macrolides with antioxidant and antiangiogenic properties, were isolated from *Streptomyces* sp. BA01, obtained from the gut of the wood ant *Formica yessensis* [9]. Another notable discovery includes 16 new formicamycins, a group of pentacyclic polyketides, produced by *Streptomyces formicae* isolated from the African ant *Tetraponera penzigi* [10]. These compounds exhibited potent antimicrobial activity against methicillin‐resistant *Staphylococcus aureus* (MRSA) and vancomycin‐resistant enterococci (VRE). In a previous study, we reported the isolation of actinomycetes from ant samples collected in Thailand [5]. Among these, *Streptomyces* strain LKA04, isolated from the ant *Polyrhachis laevissima*, showed potent antimicrobial activity. In the present study, we report for the first time the purification and characterization of the antimicrobial compound produced by this strain. In addition, we evaluated the production of the identified compound under different culture conditions.

## 2. Materials and Methods

### 2.1. Microorganisms

*Streptomyces* strain LKA04 was isolated from the ant *P*. *laevissima* [5]. The antimicrobial activity of this strain was evaluated against a panel of test microorganisms selected to represent clinically relevant gram‐positive and gram‐negative bacteria, including drug‐resistant and foodborne pathogens, as well as representative yeasts and filamentous fungi, in order to assess the antimicrobial spectrum of the strain. The test microorganisms included *Staphylococcus aureus* ATCC 25923, MRSA DMST 20646, *Bacillus subtilis* ATCC 6633, *Klebsiella pneumoniae* ATCC 13883, *Escherichia coli* ATCC 25922, *Kocuria rhizophila* ATCC 9341, *Acinetobacter baumannii* DMST 10437, *Enterococcus faecalis* DMST 4736, *Listeria monocytogenes* DMST 23145, *Salmonella typhi* DMST 21864, *Salmonella typhimurium* DMST 423, *Candida albicans* ATCC 10231, *Candida glabrata* TISTR 5206, and *Aspergillus niger* DMST 15538.

### 2.2. Characterization of Strain LKA04

#### 2.2.1. Phenotypic Properties

Cultural characteristics were observed using International *Streptomyces* Project No. 2 (ISP2) [11], following incubation of the strain at 30°C for 14 days. Microscopic morphology was examined using scanning electron microscopy after growth on ISP2 agar under the same conditions. Biochemical characteristics were assessed using standard methods [12]. Carbon utilization was determined using ISP9 medium supplemented with 1% (*w*/*v*) of a single carbon source including namely sucrose, rhamnose, raffinose, glucose, fructose, mannitol, arabinose, or xylose. ISP9 supplemented with glucose and without any carbon source served as positive and negative controls, respectively.

#### 2.2.2. Genome Features of Strain LKA04

The genome of strain LKA04 was sequenced using the Illumina MiSeq platform with 2 × 250 − bp paired‐end reads. Quality assessment of the raw sequencing data was performed using FASTQC (Babraham Bioinformatics), and adapter sequences along with low‐quality reads with a Phred quality score below Q20 were removed using the FASTP tool. Genome assembly and functional annotation was carried out through the PATRIC web service platform [13]. The resulting whole‐genome shotgun sequence was deposited in the DDBJ/ENA/GenBank database under the Accession Number JBLUUE000000000. Phylogenomic analysis of strain LKA04 was conducted using the TYGS server (http://tygs.dsmz.de), which utilizes whole‐genome sequence data [14]. The G + C content was determined from the complete genome sequence. Digital DNA–DNA hybridization (dDDH) values were calculated using the Genome‐to‐Genome Distance Calculator (GGDC) available through the DSMZ web server (http://ggdc.dsmz.de) [15]. Average nucleotide identity (ANI) was computed using JSpeciesWS (https://jspecies.ribohost.com) [16]. The identification of biosynthetic gene clusters (BGCs) responsible for secondary metabolite production was performed using antiSMASH Version 8.0.0 [17].

### 2.3. Fermentation and Isolation of Antimicrobial Compound

To prepare the inoculum, strain LKA04 was cultured in 150 mL of ISP2 broth (pH 7.0) in a 500‐mL Erlenmeyer flask at 30°C under shaking conditions at 180 rpm for 4 days. Subsequently, 1 mL of the culture was transferred into a fresh 150 mL of ISP2 broth in another 500‐mL Erlenmeyer flask. A total of 2.85 L of culture was prepared for each fermentation batch and incubated at 30°C with shaking at 180 rpm for 14 days. Following incubation, the culture broth was harvested, and secondary metabolites were extracted by partitioning the broth with an equal volume of ethyl acetate (1:1, *v*/*v*), repeated three times. The ethyl acetate layers were combined and evaporated to dryness, yielding a crude extract for subsequent antimicrobial compound isolation. The antimicrobial compound was then purified from the crude extract by bioassay‐guided fractionation using Sephadex LH‐20 open‐column chromatography with methanol as the eluent, followed by medium‐pressure liquid chromatography (MPLC) on silica gel using acetone: hexane (6:4, *v*/*v*) as the mobile phase.

The antimicrobial compound was then purified from the crude extract using various chromatographic techniques, guided by bioassay‐based fractionation.

### 2.4. Identification of Antimicrobial Compound

The crude extracts were analyzed using high‐performance liquid chromatography (HPLC) equipped with a C18 reversed‐phase column (PuropherStar; Merck, Germany, 5 *μ*m, 2.1 × 50 mm). A linear gradient system ranging from 0% to 100% acetonitrile (CH_3_CN) in water containing 0.05% formic acid was applied over 20 min. Detection was performed using a UV‐Vis detector. The resulting chromatographic profiles were compared against an in‐house database for preliminary compound identification. In addition, mass spectrometry (MS) (Agilent 6540 UHD Accurate‐Mass Q‐TOF mass spectrometer (Santa Clara, California, United States) and nuclear magnetic resonance spectroscopy (Bruker Advance NEO 400‐MHz NMR spectrometer; Karlsruche, Germany) were used to elucidate the chemical structures of the isolated secondary metabolites.

### 2.5. Production of Bioactive Compound

To evaluate the effect of different culture media on actinomycin D production, a single loop of *Streptomyces parvulus* LKA04 was obtained from a slant culture and inoculated into a 500‐mL Erlenmeyer flask containing 150 mL of ISP2 broth. The culture was incubated at 30°C with agitation at 180 rpm for 3 days to facilitate initial growth. After incubation, 1 mL of the culture was transferred to a centrifuge tube and centrifuged at 14,489 × g for 2 min. The supernatant was discarded, and the cell pellet was washed twice with deionized water to remove residual media components. The washed pellet was then resuspended in 1 mL of 0.85% NaCl solution, briefly vortexed, and inoculated into 500‐mL Erlenmeyer flasks containing 150 mL of different culture media, namely glutamic acid–histidine–fructose–mineral salts medium [18], production medium No. 54 (2% soluble starch, 0.5% glycerol, 1.0% defatted wheat germ, 0.3% meat extract, 0.3% dry yeast, and 0.3% CaCO_3_ [adjusted to pH 7.0 before sterilization]) [19], yeast extract–malt extract (ISP2) medium [11], and a modified ISP2 medium (fructose 4 g, malt extract 10 g, yeast extract 4 g, distilled water 1000 mL, pH 7.0–7.2). Each flask was incubated at 30°C on a rotary shaker at 180 rpm for 14 days. One milliliter of culture supernatant was collected every 24 h during the incubation period for HPLC analysis. The actinomycin D in the collected samples was analyzed by HPLC using a C18 column (PuropherStar; Merck, Germany, 5 *μ*m, 2.1 × 50 mm). Separation was performed under a linear gradient system ranging from 10% to 100% acetonitrile (CH_3_CN) in water at a flow rate of 0.8 mL/min over 20 min. Actinomycin D was identified based on a retention time (tR) of 18.04 ± 0.03 min. Three independent experiments were performed.

### 2.6. Antimicrobial Activity of the Compound

Antimicrobial activity was determined using the agar disc diffusion method [20] to evaluate the antimicrobial spectrum of the purified compound. Briefly, bacterial strains were cultured on Mueller–Hinton agar (MHA) at 37°C for 24 h, whereas yeast and mold were cultured on Sabouraud dextrose broth (SDB) at 30°C for 48 and 72 h, respectively. To prepare the inoculum, bacterial and yeast cultures were suspended in 0.85% NaCl and adjusted to a turbidity equivalent to 0.5 McFarland standard. The suspensions were then swabbed evenly onto the surface of MHA plates. For fungal testing, a spore suspension of *A*. *niger* was adjusted to a concentration of 1 × 10^6^ spores/mL, and 100 *μ*L of the suspension was added to melted SDB agar for use in the pour plate method. The purified compound was prepared in methanol at a concentration of 30 mg/mL, and 20 *μ*L of the solution was loaded onto a sterile paper disc. The discs were air‐dried in a clean bench for 15 min to allow methanol evaporation before being placed on the inoculated agar plates. The plates were incubated at 37°C for 18–24 h for bacterial strains, and at 30°C for 48 h for yeasts and molds. Following incubation, the diameters of the inhibition zones were measured and recorded. A disc loaded with 20 *μ*L of methanol served as the negative control in this assay.

The purified compound was prepared in methanol at a concentration of 30 mg/mL, and 20 *μ*L of the solution was loaded onto a sterile paper disc.

## 3. Results

**Figure 1:**
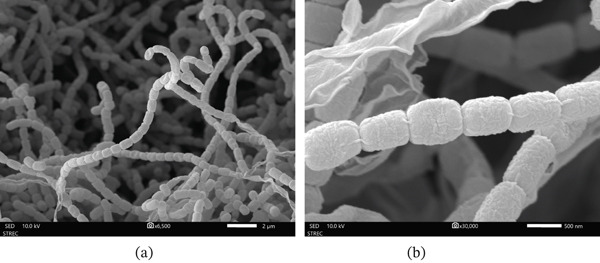
Scanning electron micrographs of *Streptomyces* strain LKA04. (a) Rectiflexibile spore chains observed after growth on ISP2 agar at 30°C for 14 days. (b) Surface morphology of individual spores showing cylindrical shape with rugose texture.

The phenotypic characterization of strain LKA04 showed positive results for starch hydrolysis and skim milk peptonization, whereas negative results were observed for nitrate reduction, gelatin liquefaction, and skim milk coagulation. The strain utilized sucrose, rhamnose, raffinose, glucose, and fructose as sole carbon sources, but was unable to utilize mannitol, arabinose, or xylose. On ISP2 medium, the aerial mycelium exhibited a gray tone, whereas the substrate mycelium appeared moderate olive brown and produced a yellow to orange soluble pigment. At the mature stage, long chains of rectiflexibile spores were observed. The spores were cylindrical in shape with a rugose surface texture (Figure 1).

**Figure 2:**
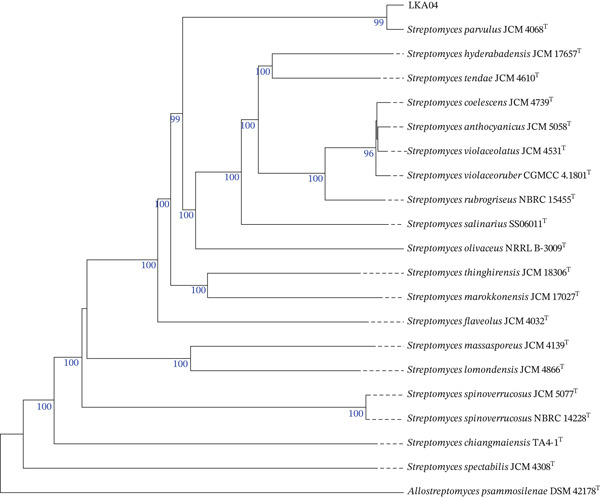
A whole‐genome sequence tree was generated with TYGS of strains LKA04 with the closely related type strains. The numbers above the branches indicate GBDP pseudobootstrap support values greater than 60% based on 100 replications. The tree is rooted at the midpoint.

The draft genome of strain LKA04 comprised 7,567,199 bp, with a DNA G + C content of 72.8% and 6860 coding sequences (CDSs). The assembly had an N50 value of 290.1 kb and consisted of 71 contigs. In addition, the genome contained 2 rRNA genes and 77 tRNA genes. CheckM analysis indicated that the genome had 96.97% completeness and 0.76% contamination, demonstrating that the assembly was of sufficient quality for further analysis. Based on the phylogenomic analysis, strain LKA04 clustered within the same node as *S*. *parvulus* JCM 4068^T^, with a bootstrap support value of 99 (Figure 2). The average nucleotide identity based on BLAST (ANIb) and MUMmer (ANIm) between strain LKA04 and *S. parvulus* JCM 4068^T^ were 99.0% and 99.19%, respectively, whereas dDDH value was 93.1% (Table S1)—all exceeding the accepted thresholds for species delineation [21]. Based on both phenotypic characteristics and genomic relatedness, strain LKA04 can be confidently identified as *S*. *parvulus*.

**Figure 3:**
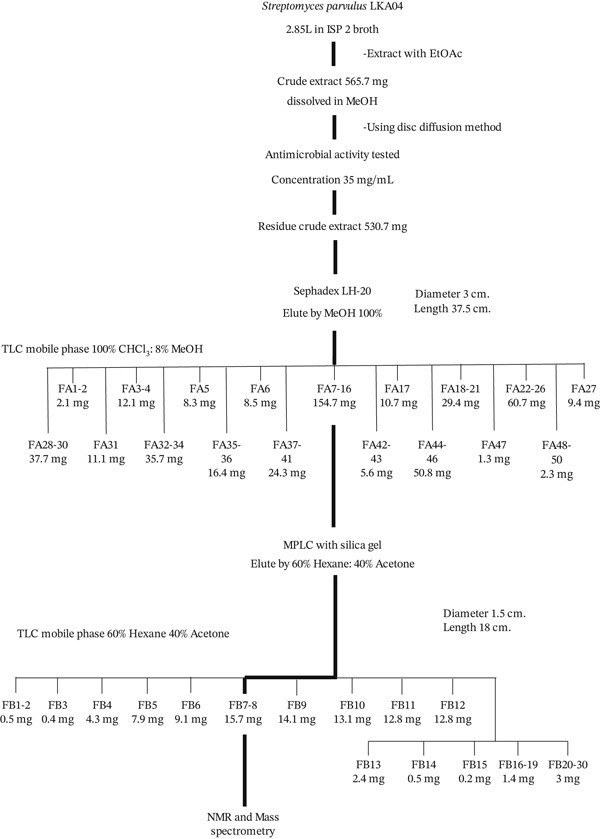
Isolation scheme of the bioactive compound produced by strain LKA04.

In this study, a total of 565.7 mg of crude ethyl acetate extract was obtained from 2.85 L of culture broth of strain LKA04. Preliminary antimicrobial screening revealed that the crude extract (35 mg/mL) exhibited inhibitory activity against *B. subtilis*, *S. aureus*, *K. rhizophila*, *S. epidermidis*, MRSA, and *Candida guilliermondii* (data not shown). To isolate the antimicrobial compound from the crude extract, 530.7 mg of the extract was dissolved in methanol and subjected to fractionation using a Sephadex LH‐20 open column (3 cm diameter × 37.5 cm length), eluted with 100% methanol to obtain 50 fractions. A bioassay‐guided screening was conducted to identify active fractions, revealing that fractions FA7–16 exhibited antimicrobial activity and were selected for further purification. These active fractions were pooled and subjected to MPLC using a silica gel column (1.5 × 18 cm), eluted with acetone:hexane (6:4, *v*/*v*), yielding 30 fractions. Antimicrobial activity testing identified fractions FB7–8 as containing the pure active compound, yielding 15.7 mg (Figure 3).

**Figure 4:**
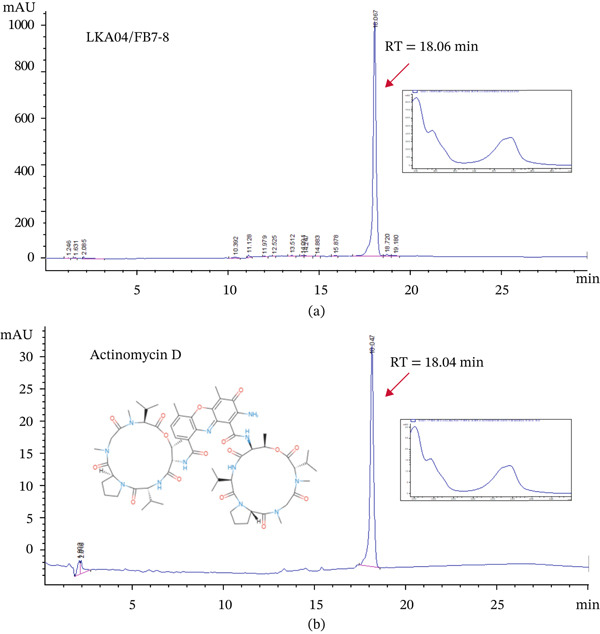
HPLC chromatograms of (a) compound FB7–8 isolated from strain LKA04 and (b) actinomycin D standard. The UV spectra of the peaks at retention times 18.06 and 18.04 min are shown in the boxes adjacent to each chromatogram.

Based on BLAST analysis using antiSMASH Version 8.0.0, several types of BGCs associated with secondary metabolite production were detected in the genome of strain LKA04. These included BGCs for siderophores, Class III lanthipeptides, hydrogen cyanide, Type II polyketide synthases (T2PKS) and Type III polyketide synthases (T3PKS), terpenes, non‐alpha poly‐amino acids (NAPAA), indoles, NRP‐metallophores, NRPS, and NRPS‐like compounds. Among these, one NRPS‐like BGC showed medium similarity to the known gene cluster responsible for actinomycin D biosynthesis, a well‐known anticancer antibiotic (Table S2 and Figure S1). However, compound FB7–8 had already been isolated as the major bioactive compound through bioassay‐guided fractionation. The detection of this NRPS‐like BGC provided genomic support suggesting that strain LKA04 may produce an actinomycin‐like compound. Therefore, compound FB7–8 was subjected to HPLC analysis and compared with an in‐house actinomycin D chromatographic reference to verify its identity. Under the HPLC conditions used in this study, compound FB7–8 was eluted at a tR of 18.06 ± 0.03 min, whereas the actinomycin D standard eluted at 18.04 ± 0.03 min. Both chromatograms exhibited similar UV absorption spectra, with characteristic peaks at 240–256 nm and 442–444 nm (Figure 4). The chemical structure of compound FB7–8 was confirmed using NMR and MS. The high‐resolution electrospray ionization mass spectrum (HRESI‐MS) of the compound showed a pseudomolecular ion [M + H]^+^ at *m*/*z* 1255.6375 (Figure S2), corresponding to the molecular formula C_62_H_86_N_12_O_16_. Furthermore, the ^1^H and ^13^C NMR spectra (Table S3 and Figures S2, S3, and S4) were consistent with those previously reported for actinomycin D by Wang et al. [22]. Based on these results, compound FB7–8 was identified as actinomycin D.

**Figure 5:**
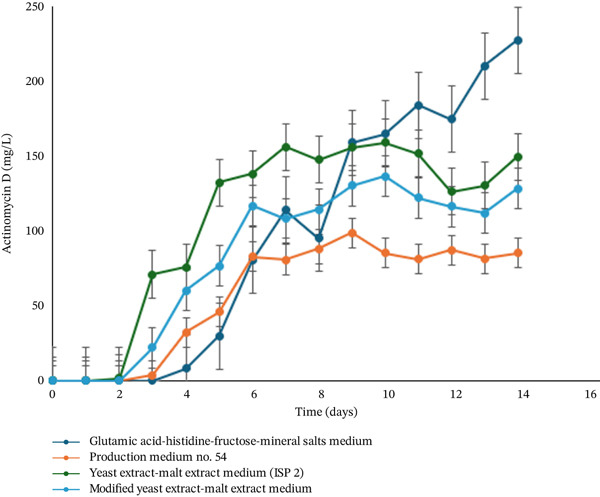
Production of actinomycin D by *Streptomyces parvulus* LKA04 cultured in four different media over a 14‐day fermentation period.

To evaluate the production of actinomycin D by strain LKA04, four different culture media were tested in this study. HPLC analysis revealed that actinomycin D was detectable after 3 days of cultivation in medium No. 54, ISP2, and modified ISP2, whereas detection in glutamic acid–histidine–fructose mineral salts medium occurred after 4 days. After 7 days of cultivation, the highest actinomycin D yield was observed in ISP2 broth (131 mg/L), followed by modified ISP2 (111 mg/L), medium No. 54 (79 mg/L), and glutamic acid–histidine–fructose mineral salts medium (77 mg/L). In ISP2, modified ISP2, and medium No. 54, the actinomycin D concentration remained relatively stable after Day 7 through the end of the 14‐day culture period. Interestingly, in the glutamic acid–histidine–fructose mineral salts medium, actinomycin D production continued to increase beyond Day 7, reaching a maximum yield of 217 mg/L by day 14 (Figure 5).

Compound FB7–8 exhibited broad‐spectrum antimicrobial activity against a range of tested microorganisms. It showed inhibitory activity against Gram‐positive bacteria, including *S. aureus*, MRSA, *B. subtilis*, *K. rhizophila*, *E. faecalis*, and *L. monocytogenes*. It also demonstrated activity against gram‐negative bacteria such as *K. pneumoniae* and *A. baumannii*, as well as the yeasts *C. albicans* and *C. glabrata*. However, no antimicrobial activity was observed against *E. coli*, *S. typhi*, *S. typhimurium*, or the fungus *A. niger* (Table 1). No reference antibiotic discs were included because the aim of the assay was not to compare the purified compound with standard antimicrobial agents, but rather to assess its antimicrobial profile against the selected microorganisms. The inhibition zones presented in Table 1 are reported as measured values from a preliminary agar disc diffusion assay for comparative evaluation only and were not interpreted according to clinical susceptibility guidelines.

**Table 1:** Antimicrobial activity of the compound FB7–8 against tested microorganisms: —, no inhibitory zone; nd, not determined.

Tested microorganisms	Inhibition zone (mm)
Gram‐positive bacteria	
*S. aureus*	24.45
MRSA	24.60
*B. subtilis*	23.70
*K. rhizophila*	35.00
*E. faecalis*	26.80
*L. monocytogenes*	23.30
Gram‐negative bacteria	
*E. coli*	—
*K. pneumoniae*	15.40
*A. baumannii*	15.00
*S. typhi*	—
*S. typhimurium*	—
Fungi	
*C. albicans*	13.30
*C. glabrata*	13.50
*A. niger*	—

## 4. Discussion

Insects have been recognized as a promising source of microorganisms with the potential to produce antimicrobial compounds with antibiotic properties. In our previous study, several ant species were found to harbor actinomycetes exhibiting notable antimicrobial activity [5]. In this present study, the identification of strain LKA04 as *S*. *parvulus* is supported not only by its phenotypic characteristics but also by genome‐based analyses, including ANI and dDDH values, which provide robust evidence for species assignment. This taxonomic placement was further supported by phylogenomic analysis, in which strain LKA04 clustered with the type strain of *S*. *parvulus* within the same clade.

*S. parvulus* has previously been isolated from a variety of environments, including soil, marine sediments, marine sponges, and lichens [23–26]. It has also been reported as an endophytic bacterium in the roots of *Aloe vera* and *Abutilon indicum* [27, 28]. Its recovery from the ant *P*. *laevissima* in the present study further broadens the known ecological distribution of this species and highlights the potential of insect‐associated habitats as reservoirs of bioactive actinomycetes.

Based on phenotypic observations, strain LKA04 produced the yellow to orange pigment on the agar media used in this study. This pigmentation may be associated with actinomycin D production, as this compound was identified as the major bioactive metabolite produced by the strain. In addition, the BGC responsible for actinomycin D production was detected in the genome of strain LKA04. The presence of a similar BGC in another publicly available *S. parvulus* genome suggests that the genetic potential for actinomycin D biosynthesis may be relatively common within this species.

Several studies have reported the broad‐spectrum antimicrobial activity of *S. parvulus*, primarily attributed to its ability to produce actinomycin D [27, 28]. Numerous studies have also focused on optimizing the culture conditions for actinomycin D production by *S. parvulus*. Sousa et al. [29] developed a chemically defined medium consisting of D(+)fructose, L(−)threonine, K_2_HPO_4_, MgSO_4_·7H_2_O, ZnSO_4_·7H_2_O, CaCl_2_·2H_2_O, FeSO_4_·7H_2_O, and deionized water to cultivate *S. parvulus* strain DAUFPE 3124, achieving a yield of 133 mg/L. In a subsequent study, the same strain was cultivated in a bioreactor under optimized conditions, yielding up to 1530 mg/L of actinomycin D [30].

Williams and Katz [18] have optimized a different chemically defined medium containing D‐fructose, L‐glutamic acid, L‐histidine, K_2_HPO_4_, MgSO_4_·7H_2_O, ZnSO_4_·7H_2_O, CaCl_2_·2H_2_O, FeSO_4_·7H_2_O, CoCl_2_·6H_2_O, and deionized water, from which they obtained yields of 500–600 mg/L using *S. parvulus* ATCC 12434. In comparison, the highest yield obtained in our study was 217 mg/L using the same medium composition described by Williams and Katz [18], although this yield was approximately three times lower than that reported for strain ATCC 12434. Variations in actinomycin D yield between studies may be attributed to differences in fermentation conditions, culture systems, and strain‐specific metabolic capabilities.

*Streptomyces parvulus* is known to produce not only actinomycin D but also actinomycin X_0_ *β*. According to the study by Chandrakar and Gupta, the strain *S. parvulus* Av‐R5 could produce both actinomycin D and actinomycin X_0_ *β* at a yield of 400 mg/L when cultured in glucose–soybean meal broth medium [27].

Actinomycin D is a potent anticancer antibiotic that exhibits cytotoxic activity against a variety of tumors, including Wilms′ tumor and rhabdomyosarcoma. In addition to its anticancer properties, actinomycin D displays broad‐spectrum antimicrobial activity against gram‐positive bacteria, as well as select gram‐negative bacteria and fungi. It has also been identified as a potential quorum sensing inhibitor. While *S. parvulus* is one of the primary producers of actinomycin D, this compound is also produced by several other *Streptomyces* species, including *Streptomyces costaricanus* [31], *Streptomyces smyrnaeus* [32], *Streptomyce heliomycini* [22], *Streptomyces hydrogenans* [33], *Streptomyces justiciae* [34] and *Streptomyces sindenensis* [35].

## 5. Conclusions

The results of this study support the identification of actinomycete strain LKA04, isolated from the ant *P*. *laevissima*, as *S*. *parvulus*. Based on the bioassay‐guided fractionation results, the antimicrobial activity exhibited by this strain was attributed to the production of the secondary metabolite actinomycin D. The isolated compound demonstrated broad‐spectrum antimicrobial activity against gram‐positive bacteria, selected gram‐negative bacteria, and fungi, consistent with findings reported in previous studies. Among the tested media, the highest yield of actinomycin D (217 mg/L) was achieved when *S. parvulus* LKA04 was cultured in glutamic acid–histidine–fructose mineral salts broth at 30°C under shaking conditions (180 rpm) for 14 days. These findings highlight the potential of ant‐derived actinomycetes as a promising source of bioactive compounds. Continued investigation into these microorganisms is warranted to further explore their potential in drug discovery and development.

## Author Contributions

T.T.: experiments and draft manuscript writing; W.J. and Y.S.: ant sample collection and identification; C.C.: formal analysis; A.S.: experiments; S.T.: conceptual advice; W.P.: funding acquisition, experimental design, formal analysis, research summary, and manuscript reviewing and editing.

## Funding

This study was supported by the Thailand Science Research and Innovation Fund of Chulalongkorn University (HEA_FF_68_163_3300_010).

## Ethics Statement

The *Polyrhachis laevissima* specimens examined in this study were collected incidentally from a nonprotected, community‐accessible area. As the species is not classified under protected wildlife categories, no specific collection permit or formal ethical approval was required under the relevant regulations in force at the time of sampling.

## Conflicts of Interest

The authors declare no conflicts of interest.

## Supporting information


**Supporting Information** Additional supporting information can be found online in the Supporting Information section. Table S1 ANIb and ANIm values (%) and the digital DNA‐DNA hybridization (dDDH) values between the draft genomes of strain LKA04 and its related *Streptomyces* type strain. Table S2: Identified secondary metabolite biosynthetic gene clusters (BGCs) in the genome of strain LKA04, based on BLAST search using antiSMASH Version 8.0.0. Only BGCs with medium to high similarity confidence are shown. Table S3: ^1^H NMR and ^13^C NMR data of FB7–8: Actinomycin D (CDCl_3_, ^1^H: 400 MHz, ^13^C: 100 MHz). Figure S1: Comparison of biosynthetic gene clusters (BGCs) detected in the genome of strain LKA04. (a) Predicted BGCs in strain LKA04 associated with actinomycin D biosynthesis. (b) Reference BGC of the actinomycin D biosynthetic gene cluster from *Streptomyces anulatus*. (c) Chemical structure of actinomycin D. Figure S2: The ESI‐MS of compound FB7–8. Figure S3: ^1^H NMR spectrum of compound FB7–8 (400 MHz, CDCl_3_). Figure S4: ^13^C NMR spectrum of compound FB7–8 (100 MHz, CDCl_3_).

## Data Availability

The data that support the findings of this study are openly available in *Streptomyces* sp. (LKA04) at https://www.ncbi.nlm.nih.gov/genbank/ (Reference Number PRJNA1185696).
