# Supplementary material for: Characterization and Production of Antimicrobial Compound by Streptomyces Isolated From the Ant Polyrhachis laevissima
Source: Int J Microbiol. 2026 May 22;2026:8925797. doi: 10.1155/ijm/8925797 (PMC13195440; doi:10.1155/ijm/8925797)

Table S1 ANIb and ANIm values (%) and the digital DNA-DNA hybridization (dDDH) values between the draft genomes of strain LKA04 and its related *Streptomyces* type strain

strain	Genome size (bp)	Assembly accession number	%GC Content	%dDDH	ANIb	ANIm
LKA04	7567199	GCA_047979815	72.8	-	-	-
Streptomyces parvulus JCM 4068	7693235	GCA_014648855	72.8	93.1	99.0	99.19

Table S2. Identified secondary metabolite biosynthetic gene clusters (BGCs) in the genome of strain LKA04, based on BLAST search using antiSMASH version 8.0.0. Only BGCs with medium to high similarity confidence are shown.

Cluster	Type	Most similar known cluster (class)	Similarity Confidence
1	Siderophore	Desferrioxamin B/ desferrioxamine E	High
2	Lanthipeptide-class iii, hydrogen-cyanide	SapB	High
3	T2PKS	Spore pigment	Medium
4	Terpene	albaflavenone	High
5	NAPAA, terpene	Isorenieratene	High
6	Indole	5-dimethylallylindole-3-acetonitrile	High
7	T3PKS	germicidin	High
8	NRP-metallophore, NRPS	Coelichelin	High
9	ectoine	Ectoine	High
10	NRPS-like	Actinomycin D	Medium
11	Terpene	Geosmin	High
12	Terpene	Hopene	Medium

Table S3. ^{1}H NMR and ^{13}C NMR data of FB7-8: Actinomycin D ($CDCl_3$, ^{1}H: 400 MHz, ^{13}C: 100 MHz)

	Ring-A			Ring-B		
	Position	δ_C, type	δ_H (J in Hz)	Position	δ_C, type	δ_H (J in Hz)
Thr	1	168.98, C		1	168.50, C	
	2	55.15, CH	4.619 (dd)	2	54.79, CH	4.51 (dd)
	3	75.03, CH	5.2 (m)	3	74.94, CH	5.2 (m)
	4	17.31, CH_3	1.246 (s)	4	17.72, CH_3	1.235 (s)
	NH		7.13 (d, 6.8)	NH		7.70 (d, 6.4)
Val	1	173.63, C		1	173.27, C	
	2	58.87, CH	3.56 (m)	2	58.72, CH	3.56 (m)
	3	31.78, CH	2.12 (m)	3	31.53, CH	2.12 (m)
	4	19.26, CH_3	1.12 (d, 6)	4	19.08, CH_3	1.12 (d, 6)
	5	19.02, CH_3	0.9 (d, 6.8)	5	18.96, CH_3	0.88 (d, 6.8)
	NH		8.21 (d, 5.6)	NH		8.05 (d, 6)
Pro	1	173.24, C		1	173.24, C	
	2	56.47, CH	5.99 (d, 9.2)	2	56.24, CH	5.92 (d, 9.2)
	3	30.94, CH_2	1.85 (m), 2.65 (m)	3	31.23, CH_2	1.81 (m), 2.65 (m)
	4	22.99, CH_2	2.17 (m), 2.25 (m)	4	22.82, CH_2	2.16 (m), 2.25 (m)
	5	47.56, CH_2	3.716 (m)	5	47.32, CH_2	3.716 (m)
Sar	1	166.49, C		1	166.38, C	
	2	51.35, CH_2	4.81 (d, 17.6), 3.61 (d, 17.6)	2	51.35, CH_2	4.73 (d, 17.6), 3.63 (d, 17.2)
MeVal	NMe	34.93, CH_3	2.87 (s)	NMe	34.86, CH_3	2.87 (s)
	1	167.66, C		1	167.55, C	
	2	71.35, CH	2.65 (m)	2	71.16, CH	2.65 (m)
	3	26.94, CH	2.65 (m)	3	26.89, CH	2.65 (m)
	4	21.66, CH_3	0.95 (d)	4	21.56, CH_3	0.95 (d)
	5	19.22, CH_3	0.743 (s)	5	19.08, CH_3	0.727 (s)
	Nme	39.26, CH_3	2.904 (s)	Nme	39.14, CH_3	2.930 (s)
Chromophore	1	101.67, C				
	2	147.52, C				
	3	179.05, C				
	4	113.51, C				
	4a	145.06, C				
	5a	140.47, C				
	6	127.67, C				
	7	130.28, CH	7.35 (d, 8)			
	8	125.72, CH	7.61 (d, 8)			
	9	132.55, C				
	9a	129.08, C				
	10a	145.84, C				
	11	168.98, C				
	12	7.76, CH_3	2.233 (s)			
	13	15.04, CH_3	2.540 (s)			
	14	166.55, C				

Figure S1. Comparison of biosynthetic gene clusters (BGCs) detected in the genome of strain LKA04. (a) Predicted BGCs in strain LKA04 associated with actinomycin D biosynthesis. (b) Reference BGC of the actinomycin D biosynthetic gene cluster from *Streptomyces anulatus*. (c) Chemical structure of actinomycin D.

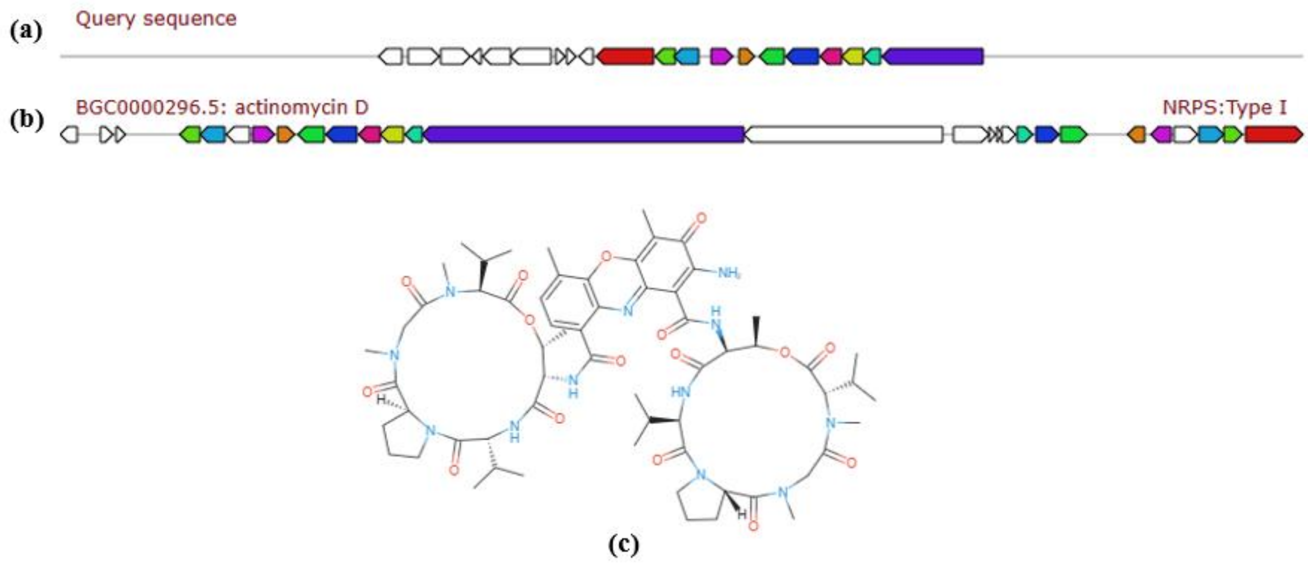

Figure S2 The ESI-MS of compound FB7-8

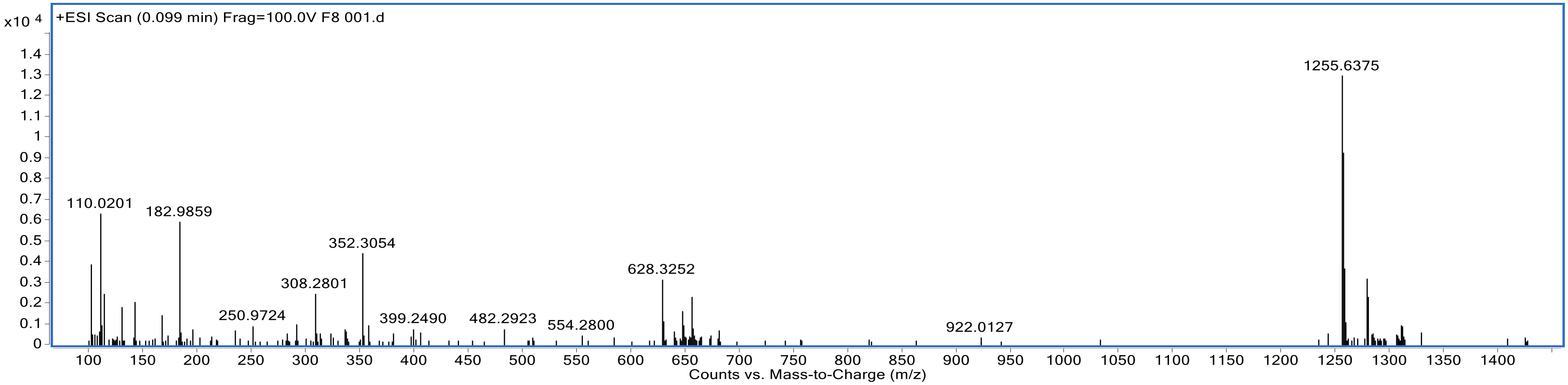

Figure S3 ^{1}H NMR spectrum of compound FB7-8 (400 MHz, $CDCl_3$)

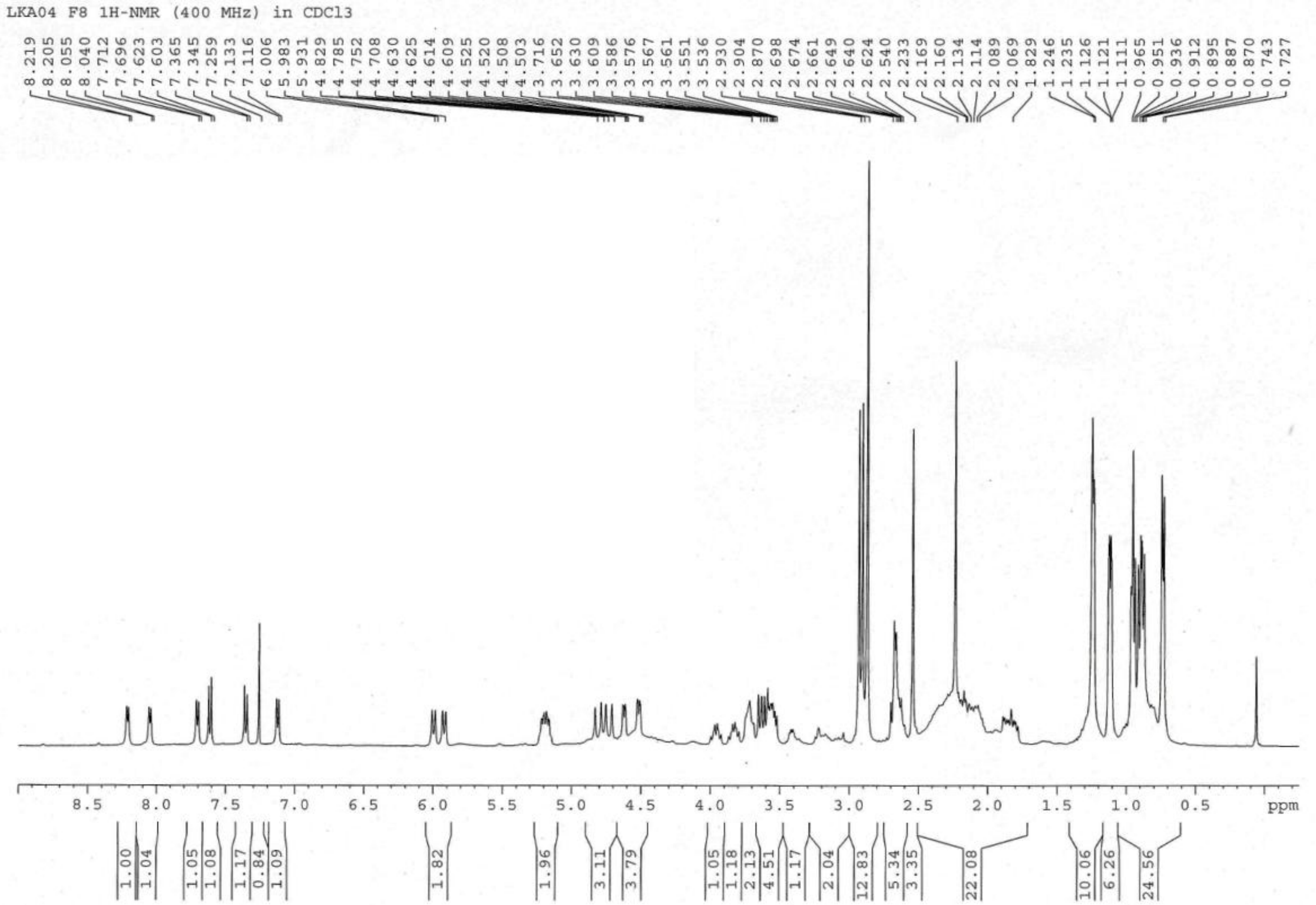

Figure S4 ^{13}C NMR spectrum of compound FB7-8 (100MHz, $CDCl_3$)

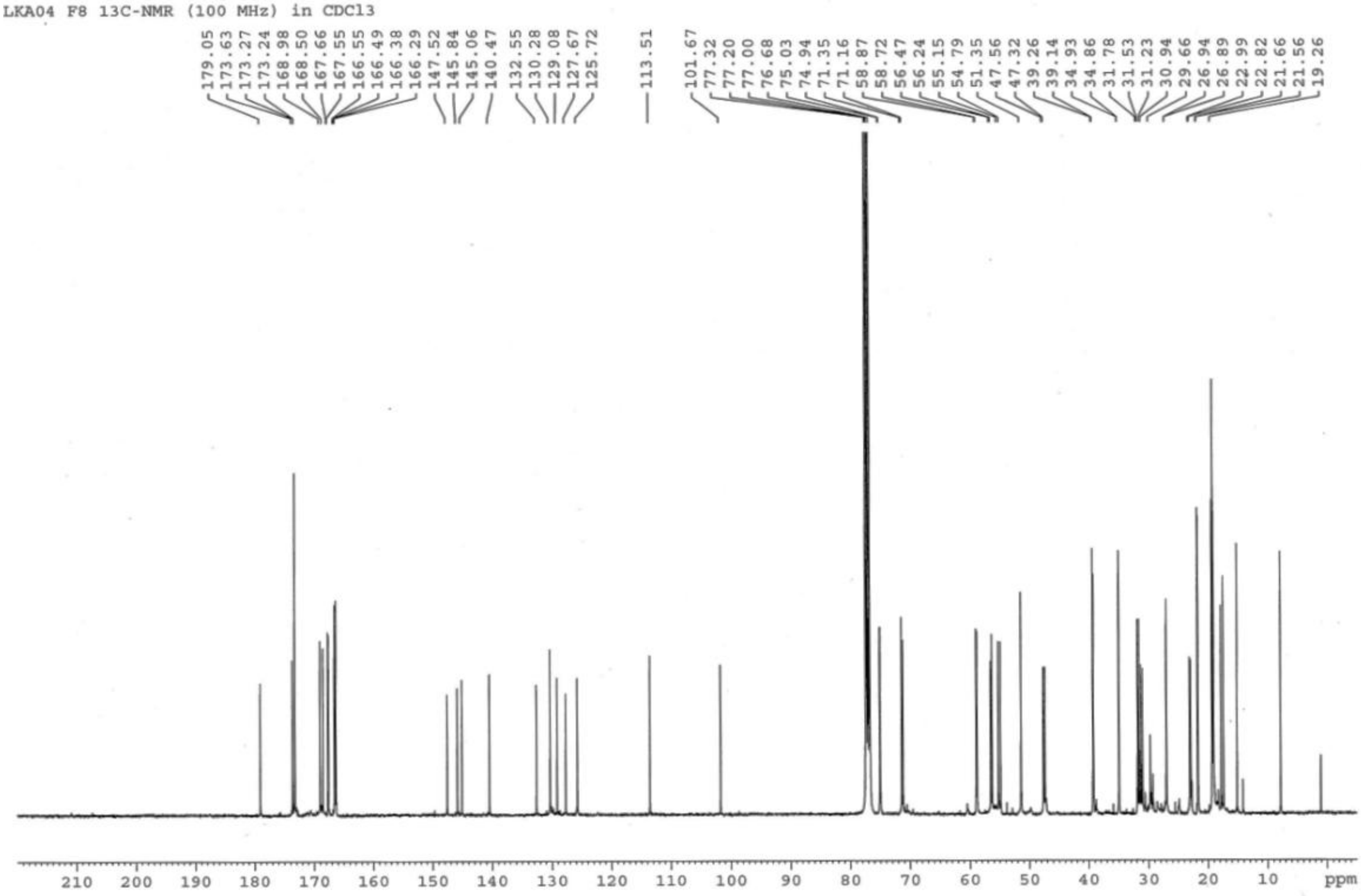

Supplement: Supplementary file 1 — Supporting Information Additional supporting information can be found online in the Supporting Information section. Table S1 ANIb and ANIm values (%) and the digital DNA‐DNA hybridization (dDDH) values between the draft genomes of strain LKA04 and its related Streptomyces type strain. Table S2: Identified secondary metabolite biosynthetic gene clusters (BGCs) in the genome of strain LKA04, based on BLAST search using antiSMASH Version 8.0.0. Only BGCs with medium to high similarity confidence are shown. Table S3: 1H NMR and 13C NMR data of FB7–8: Actinomycin D (CDCl3, 1H: 400 MHz, 13C: 100 MHz). Figure S1: Comparison of biosynthetic gene clusters (BGCs) detected in the genome of strain LKA04. (a) Predicted BGCs in strain LKA04 associated with actinomycin D biosynthesis. (b) Reference BGC of the actinomycin D biosynthetic gene cluster from Streptomyces anulatus. (c) Chemical structure of actinomycin D. Figure S2: The ESI‐MS of compound FB7–8. Figure S3: 1H NMR spectrum of compound FB7–8 (400 MHz, CDCl3). Figure S4: 13C NMR spectrum of compound FB7–8 (100 MHz, CDCl3). [file IJM-2026-8925797-s001.pdf]
